# John Edward Cooper, BA, BM (Oxon), DPM (Lond), FRCP, FRCPsych

**DOI:** 10.1192/bjb.2024.129

**Published:** 2025-10

**Authors:** Neil Nixon

Formerly Professor of Psychiatry, University of Nottingham Medical School

**Figure:**
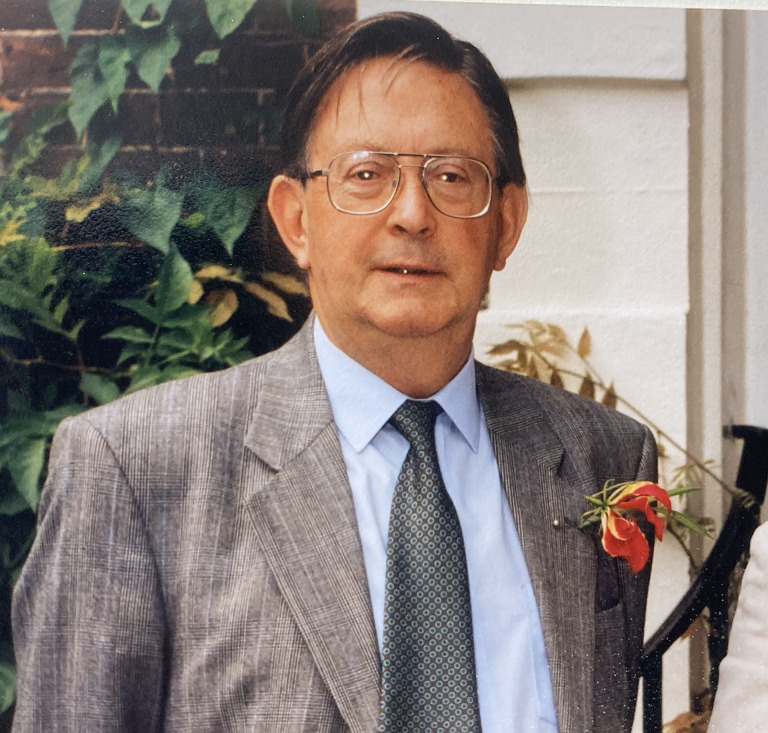

John Cooper died at the age of 95 on 11 September 2024. He made a remarkable contribution to psychiatry. While he was in training at the Maudsley Hospital in the mid-1960s, he began to collaborate in a vital era of international research – carefully illustrating the confusion caused by inconsistent diagnostic criteria and developing the foundations of our current approach. His involvement in the US-UK Diagnostic Project helped to demonstrate for the first time that international collaborative research in clinical psychiatry using standardised instruments was possible, and that it might provide a basis for improvement of standards of international classification. Understanding this potential, John developed a central role in two major World Health Organization (WHO) programmes, dealing with the operational classification of psychiatric diagnosis and with the prevalence and form of severe mental disorders, including the seminal WHO international studies on schizophrenia. He was a highly respected consultant for several other WHO programmes and helped to deliver the first operational diagnostic criteria for mental disorders, in the ICD-10.

This international perspective on mental illness remained an abiding interest that John brought to the University of Nottingham when he was appointed as the first Professor of Psychiatry at the new medical school in 1971. Under his influence, Nottingham became the UK field centre for the WHO Determinants of Outcome study (1978–1980). As an outstanding teacher – always modest about his achievements, willing to listen and to help – John inspired countless trainees, forming a respected tradition of epidemiological research in Nottingham. He was generous and progressive in his approach to clinical services, for example, supporting Margaret Oates to admit mentally ill mothers together with their babies – at that time revolutionary but now seen as best practice in perinatal psychiatry. John saw these developments within a deeper tradition in Nottingham, extending back to Duncan MacMillan's open-door policy at Mapperley Hospital. Aware of the initial ‘disbelief’ of overseas visitors that patients with psychiatric disorders could be allowed such freedom, John saw in these advances the basic importance of treating those suffering from mental disorders as human beings with the prospect of recovery – and he often commented on the role these developments had in countering another abiding concern, the stigmatisation of people with mental illness.

John was born on 27 July 1929 in Sheffield, to Cyril, a photographer, and Enid, a ballroom dancing teacher. He was younger brother to Jim and elder brother to Diana, who both survive him. John attended King Edward VII school in Sheffield and gained a scholarship to study medicine at Lincoln College, Oxford, when he was 17. At Oxford, John proved himself a gifted sportsman, winning the athletics cup at Lincoln College in 1949. After graduating in medicine, he completed his military service as a doctor in the Royal Air Force, stationed at RAF Lyneham, acting on standby for the casualty evacuation service during the Suez crisis. He then secured a place to train in psychiatry at the Maudsley Hospital in London, where he met and married Chloë Simpson in 1959. They had three children over the following decade: Elizabeth, Polly and Martin. Although John separated from Chloë in 1987 and they later divorced, he remained deeply connected to his family. Beyond this, John maintained lifelong interests in natural history, travel, anthropology, photography, astronomy, bird-watching, the world of insects and fishing, while continuing to excel at sports, including winning numerous club squash and golf trophies.

He had a keen eye for the humour of his position, observing that wherever he travelled, whether in Siberia or Bali, he would be met off the plane and immediately taken to the local mental health facility. He wrote warmly of experiences beyond this, including a picnic at Lake Baikal in Siberia, hosted by an enthusiastic Russian psychiatrist, who became ever more enthusiastic after repeated toasts of vodka – allegedly claiming that ‘a toast was sincere only if both toaster and responder took three drinks of vodka, rather than one’ – to the point of expressing her admiration of English men and finally asking for a kiss, in the name of ‘international goodwill’. John recounted that he ‘stood [his] ground as she approached’ and was relieved, through the haze of the rapidly disappearing third bottle of vodka, to receive only a simple continental expression of her affection – on his cheek. John wrote about his travels in a style which is shot through with goodwill and acceptance of the places and cultures he encountered.

Through his retirement, John remained a true internationalist, fiercely proud of the WHO approach to classifying mental health and never losing sight of the basic clarity this brought to the development of sound mental health services. In a personal dedication to John, from a text reviewing this key era of international research, Norman Sartorius, past director of the Division of Mental Health of the WHO, wrote of John's contribution to the programmes of the WHO with admiration, stating simply, ‘present at the creation; a presence throughout’.

He is survived by his three children and two grandchildren.

